# KoNA: Korean Nucleotide Archive as A New Data Repository for Nucleotide Sequence Data

**DOI:** 10.1093/gpbjnl/qzae017

**Published:** 2024-03-01

**Authors:** Gunhwan Ko, Jae Ho Lee, Young Mi Sim, Wangho Song, Byung-Ha Yoon, Iksu Byeon, Bang Hyuck Lee, Sang-Ok Kim, Jinhyuk Choi, Insoo Jang, Hyerin Kim, Jin Ok Yang, Kiwon Jang, Sora Kim, Jong-Hwan Kim, Jongbum Jeon, Jaeeun Jung, Seungwoo Hwang, Ji-Hwan Park, Pan-Gyu Kim, Seon-Young Kim, Byungwook Lee

**Affiliations:** Korea Bioinformation Center, Korea Research Institute of Bioscience & Biotechnology, Daejeon 34141, Republic of Korea; Korea Bioinformation Center, Korea Research Institute of Bioscience & Biotechnology, Daejeon 34141, Republic of Korea; Korea Bioinformation Center, Korea Research Institute of Bioscience & Biotechnology, Daejeon 34141, Republic of Korea; Korea Bioinformation Center, Korea Research Institute of Bioscience & Biotechnology, Daejeon 34141, Republic of Korea; Korea Bioinformation Center, Korea Research Institute of Bioscience & Biotechnology, Daejeon 34141, Republic of Korea; Korea Bioinformation Center, Korea Research Institute of Bioscience & Biotechnology, Daejeon 34141, Republic of Korea; Korea Bioinformation Center, Korea Research Institute of Bioscience & Biotechnology, Daejeon 34141, Republic of Korea; Korea Bioinformation Center, Korea Research Institute of Bioscience & Biotechnology, Daejeon 34141, Republic of Korea; Korea Bioinformation Center, Korea Research Institute of Bioscience & Biotechnology, Daejeon 34141, Republic of Korea; Korea Bioinformation Center, Korea Research Institute of Bioscience & Biotechnology, Daejeon 34141, Republic of Korea; Korea Bioinformation Center, Korea Research Institute of Bioscience & Biotechnology, Daejeon 34141, Republic of Korea; Korea Bioinformation Center, Korea Research Institute of Bioscience & Biotechnology, Daejeon 34141, Republic of Korea; Korea Bioinformation Center, Korea Research Institute of Bioscience & Biotechnology, Daejeon 34141, Republic of Korea; Korea Bioinformation Center, Korea Research Institute of Bioscience & Biotechnology, Daejeon 34141, Republic of Korea; Korea Bioinformation Center, Korea Research Institute of Bioscience & Biotechnology, Daejeon 34141, Republic of Korea; Korea Bioinformation Center, Korea Research Institute of Bioscience & Biotechnology, Daejeon 34141, Republic of Korea; Korea Bioinformation Center, Korea Research Institute of Bioscience & Biotechnology, Daejeon 34141, Republic of Korea; Korea Bioinformation Center, Korea Research Institute of Bioscience & Biotechnology, Daejeon 34141, Republic of Korea; Korea Bioinformation Center, Korea Research Institute of Bioscience & Biotechnology, Daejeon 34141, Republic of Korea; Korea Bioinformation Center, Korea Research Institute of Bioscience & Biotechnology, Daejeon 34141, Republic of Korea; Korea Bioinformation Center, Korea Research Institute of Bioscience & Biotechnology, Daejeon 34141, Republic of Korea; Korea Bioinformation Center, Korea Research Institute of Bioscience & Biotechnology, Daejeon 34141, Republic of Korea

**Keywords:** Korea BioData Station, Nucleotide sequence, Next-generation sequencing repository, Genomics, Deposition and access of big data

## Abstract

During the last decade, the generation and accumulation of petabase-scale high-throughput sequencing data have resulted in great challenges, including access to human data, as well as transfer, storage, and sharing of enormous amounts of data. To promote data-driven biological research, the Korean government announced that all biological data generated from government-funded research projects should be deposited at the Korea BioData Station (K-BDS), which consists of multiple databases for individual data types. Here, we introduce the Korean Nucleotide Archive (KoNA), a repository of nucleotide sequence data. As of July 2022, the Korean Read Archive in KoNA has collected over 477 TB of raw next-generation sequencing data from national genome projects. To ensure data quality and prepare for international alignment, a standard operating procedure was adopted, which is similar to that of the International Nucleotide Sequence Database Collaboration. The standard operating procedure includes quality control processes for submitted data and metadata using an automated pipeline, followed by manual examination. To ensure fast and stable data transfer, a high-speed transmission system called GBox is used in KoNA. Furthermore, the data uploaded to or downloaded from KoNA through GBox can be readily processed using a cloud computing service called Bio-Express. This seamless coupling of KoNA, GBox, and Bio-Express enhances the data experience, including submission, access, and analysis of raw nucleotide sequences. KoNA not only satisfies the unmet needs for a national sequence repository in Korea but also provides datasets to researchers globally and contributes to advances in genomics. The KoNA is available at https://www.kobic.re.kr/kona/.

## Introduction

The drastic price decrease of next-generation sequencing (NGS) data and improvement of sequencing platforms with higher data throughput have resulted in the production of more than tens of petabases of raw sequencing data [1,2]. For example, in Korea, a few petabases of NGS data in diverse biological systems have been generated from several national projects, such as the Korea Post-Genome Project. To deposit, store, and share the enormous amount of raw nucleotide sequence data, there have been many efforts to construct repository databases by national and international data centers, such as the National Center for Biotechnology Information (NCBI), the European Molecular Biology Laboratory’s European Bioinformatics Institute (EMBL-EBI), the DNA Data Bank of Japan (DDBJ), and the National Genomics Data Center (NGDC) [3–6]. These publicly available repositories provide datasets to global scientific communities and facilitate data reuse, which can reduce costs and usher advances in genomics. Despite the impressive contribution of these repositories, additional national repositories are emerging in many countries mainly for three reasons. First, many countries are carrying out nationwide genome projects and producing huge amounts of data that need to be archived. Second, domestic networks have larger upload and download bandwidths, enabling faster data transfer than international networks. Third, deposition of and access to human data are controlled by domestic laws because of the sensitive nature of human data [4,6]. The Korea BioData Station (K-BDS), which was developed by the Korea Bioinformation Center (KOBIC), is a data archive that was initiated to address these three reasons.

First, the K-BDS is a central archive for all biological data generated by Korean government-funded research projects. In 2020, the Korean government announced an initiative called the “Strategy on Biological Research Resource Big Data” to promote data-driven research in biology. The announcement of the initiative was followed by the introduction of a data policy that mandated that all biological data generated from government-funded research projects should be deposited in a central data archive, to which the K-BDS was designated [7]. Given the data policy applied to all biological data types, the K-BDS consists of several databases, each specialized in collecting and sharing a specific data type, such as NGS, microarray, quantitative proteomics, metabolomics, and bioimages. Second, the K-BDS enables significantly faster data submission and sharing within a nation. For domestic researchers in Korea, data submission and access to the K-BDS (949 Mb/s upload and 951 Mb/s download) are approximately eight times faster on average than those to the NCBI (123 Mb/s upload and 107 Mb/s download), according to the Communication Service Quality Evaluation Results of the Korea Ministry of Science and Information and Communication Technologies (ICT) in 2019 (http://www.smartchoice.or.kr/smc/info/evaluateDownload.do). Third, the K-BDS handles sensitive human data in a manner that complies with relevant Korean laws and government policies, such as the Bioethics and BioSafety Act and the Guidelines for the Use of Health and Medicine Data.

In this study, we introduce the Korean Nucleotide Archive (KoNA), a nucleotide sequence data-specific database in the K-BDS, to fulfill the unmet needs for efficient collection, management, and sharing of nucleotide sequence data in Korea. KoNA currently accepts NGS data submissions with a plan to extend it to other types of nucleotide sequences, such as assembled sequences and primer sequences. Currently, the Korean Read Archive (KRA) in KoNA contains NGS data encompassing various sequencing experiments, namely genomics, transcriptomics, epigenomics, and metagenomics, as well as diverse organisms, such as humans, animals, plants, and microorganisms. Furthermore, the KoNA is coupled to a high-speed data transmission system called GBox, which is coupled to a cloud computing service for large-scale biological data analysis called Bio-Express [8,9]. As all these services are offered in English, they can be used by researchers worldwide.

## Results

### Overview of KoNA

Designated by the “Act on the Acquisition, Management, and Utilization of Biological Research Resources”, KOBIC is a national center for collecting biological data generated by Korean government-funded research projects. In 2021, KOBIC established the K-BDS, which consists of several repository databases specific to individual data types. One such database is KoNA, which collects and provides primary nucleotide sequence data, such as FASTQ- or FASTA-formatted files generated by diverse NGS techniques and rich metadata. To prepare for international alignment with existing public databases, the data standards and structures of the following repository databases were examined: Sequence Read Archive (SRA) of NCBI, European Nucleotide Archive (ENA)/SRA of EBI, SRA of DDBJ, and Genome Sequence Archive (GSA) of NGDC [3–6].

Upon examining the existing repositories, we adopted the overall data structure implemented in these databases, which was used to hierarchically organize metadata underlying the NGS data. Specifically, we adopted BioProject and BioSample to capture descriptive information on research projects and biological samples, respectively, Experiment to capture instrument and library information, and Run to capture sequence data files. For BioSample, we used only the standard packages of the International Nucleotide Sequence Database Collaboration (INSDC) but not the MIxS packages of the Genome Standards Consortium.

**Figure 1:**
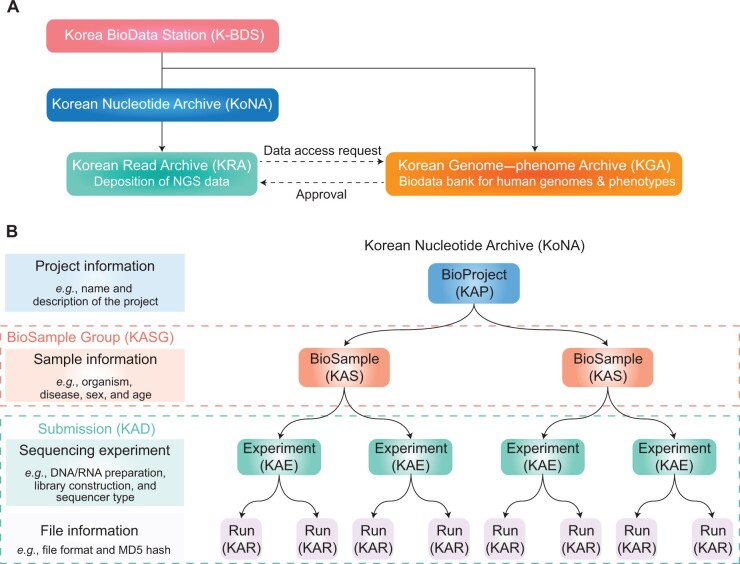
Database structure and data model of K-BDS and KoNA **A**. K-BDS and KoNA database structure. KoNA, the national nucleotide sequence repository, is one of the databases in K-BDS. KoNA contains the KRA, a primary NGS data repository. Furthermore, for human data, KoNA is systematically linked with the KGA of K-BDS, a database for human nucleotide sequences. All the primary NGS data of human samples are accessible in KRA after KGA approves data access request by users. **B**. The current data model of KoNA. It consists of four objects: BioProject, BioSample, Experiment, and Run. For each data object, description and attribute example are presented in the right panel, with prefixes of accession IDs presented in the parentheses. KAP, KAS, KAE, and KAR are the prefixes of accession IDs for BioProject, BioSample, Experiment, and Run objects in KoNA, respectively. The dashed boxes indicate the groups of BioSamples (“BioSample Group”) and Experiments and Runs (“Submission”) with “KASG” and “KAD” as the prefixes for their accession IDs, respectively. The “K” in the prefixes of all the accession IDs denotes “Korea/Korean”. KoNA, Korean Nucleotide Archive; K-BDS, Korea BioData Station; KRA, Korean Read Archive; KGA, Korean Genome–phenome Archive; NGS, next-generation sequencing.

Currently, KoNA contains only the KRA, a sequencing read data archive akin to the NCBI’s SRA, EBI’s ENA/SRA, DDBJ’s SRA, and NGDC’s GSA. KoNA is also systematically linked to the Korean Genome–phenome Archive (KGA), a repository of controlled-access data for human genotypes and phenotypes, which is similar to the European Genome–phenome Archive (EGA), the database of Genotypes and Phenotypes (dbGaP), and Japanese Genotype–phenotype Archive (JGA) (**Figure 1**A). While most data deposited in the KRA can be accessed without restrictions, human-related data in the KRA are only accessible after the approval of the data access request to the KGA operated by the Korea Human BioData Bank.

### Data model and structure of KoNA

The metadata model of KRA is equivalent to those established by the INSDC databases and the NGDC GSA, in which all the attributes are categorized into four objects (Figure 1B): study (*i.e.*, “BioProject” object with KAP prefix; *e.g.*, KAP210001), sample information (*i.e.*, “BioSample” object with KAS prefix; *e.g.*, KAS21000001), sequencing experiment (*i.e.*, “Experiment” object with KAE prefix; *e.g.*, KAE21000001), and data files (*i.e.*, “Run” object with KAR prefix; *e.g.*, KAR21000001). For the numerical part, the first two decimal digits indicate the year of the accession ID issue, and the other decimal digits are sequential numbers assigned in chronological order. We denote a group of BioSamples as the BioSample Group with a KASG prefix (*e.g.*, KASG210001) to manage and present the relevant BioSamples in the system for user convenience. We issue an accession ID for a group of Experiments and Runs annotated in one submission batch (*i.e.*, “Submission” object with KAD prefix; *e.g.*, KAD2100001).

### Data submission and quality control

**Figure 2:**
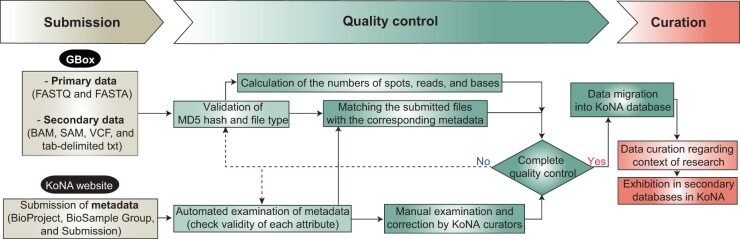
Workflow of data submission, quality control, and curation processes During the submission process, primary data (*i.e.*, raw sequence data) and/or secondary data should be submitted through GBox, whereas relevant metadata should be submitted to the KoNA website. During quality control, we confirm the MD5 hashes and file extensions of the submitted data, and their metadata undergo automated and manual examinations. Finally, all the data and metadata are migrated to KoNA, followed by data curation by the KoNA curators.

The user-friendly interface of the KoNA website helps users intuitively understand the data submission, retrieval, and access procedures and obtain the statistics of the data deposited in KoNA (*e.g.*, the numbers of deposited BioProjects, BioSample Groups, Submissions, and Runs). To deposit data in KoNA, raw sequence data and accompanying metadata must be submitted through GBox and the website, respectively (**Figure 2**). Before the metadata submission, primary raw sequence data and processed secondary data, if available, should be uploaded to the user space in GBox. For primary data, KoNA allows the FASTQ and FASTA file formats. The submission of FASTQ files is highly recommended when data are generated by short-read sequencing methods, such as the Illumina and MGI platforms, to maintain Phred quality scores for individual bases. For secondary data, KoNA allows file formats such as binary alignment map (BAM), sequence alignment map (SAM), variant call format (VCF), and tab-delimited text. Secondary data alone, without corresponding primary data, are not accepted.

As described in the submission guidelines on the website, the submission of metadata proceeds in the following order: BioProject, BioSample Group, and Submission. Online spreadsheets with attribute names previously established by the INSDC provide the details of multiple BioSamples in a BioSample Group and those of multiple Experiments and Runs in a Submission. Moreover, to match individual Experiments with the primary and secondary data uploaded to GBox, the file names and directories in the GBox user space should be described in the submission spreadsheet.

We aimed to collect high-quality data to increase data usability. Therefore, we successively perform quality control for both primary and secondary data, followed by the metadata. For primary and secondary data, we first automatically validate the MD5 hashes and confirm the file formats or extensions with those input into the corresponding metadata spreadsheet. For each primary dataset, the number of spots, reads, and bases are assessed using in-house scripts. By contrast, metadata submitted to the KoNA website are first subjected to an automated examination to verify the integrity of each attribute in the metadata. Currently, the automated examination includes a checkup of whether mandatory fields were entered and whether correct variable types, such as numeric, character, and date, were entered for each attribute. Further development is underway to expand the functionality of automated examinations. The automated examination is followed by manual examination and correction by KoNA curators who are data experts at KOBIC. The curators communicate closely with the submitters, examine the research context, and correct the metadata to fill in all the critical content required for data reanalysis and convey the original biological contexts of the submitted data to data users. Upon completion of the quality control processes, the submitted data are migrated to the KoNA/KRA databases with accession IDs for the individual objects. Finally, KoNA curators group the deposited studies into data collections based on their research contexts during the data curation processes. All curated collections are planned to be shown in the secondary databases of the KoNA. To aid data submission, user documentation in English is provided, and a helpdesk is available; contact information is listed on the Contact Us webpage.

### Data retrieval and access

For data deposited in KoNA, all publicly accessible BioProjects are listed in the “Browse” menu. Moreover, users can search for relevant BioProjects, BioSample Groups, and Submissions using keyword queries. To enable fast and flexible responses to a given query, we applied a search engine for big data based on Elasticsearch [10] and developed a search engine optimized for the KoNA system. The KoNA system has the following advantages. First, it stores indexed data and is fully searchable in near real time. Second, it supports a high-speed search for structured and unstructured data using a big data platform capable of parallel distributed processing. Third, it supports the flexible extension of diverse data types using schemaless NoSQL. Fourth, it supports diverse programmatic access methods by providing RESTful Application Programming Interface (API). Finally, it visualizes real-time statistics from access logs using the Elasticsearch and Kibana tools.

Among the 666 Submissions deposited in the KRA database, 602 Submissions (90.4%) were released by July 2022. Journal reviewers and editors can access embargoed Submissions if the submitters issue access tokens to their data on the KoNA website. Users authorized by the KGA can access 117 released Submissions out of the 137 controlled-access Submissions. The data request and access procedures in the KGA are in accordance with Korea’s “Bioethics and BioSafety Act” and related ethical restrictions. Submissions with nonhuman data are readily accessible to the public.

### Data transfer and cloud-based data processing utilities

KoNA was designed as a package deal in which users not only deposit and provide their nucleotide sequence data but also enhance their data experience by tying KoNA together with a fast and reliable data transfer system and diverse genome analysis utilities. To this end, KoNA adopts a high-speed data transmission system called GBox, which can transfer massive sequence data nine times faster than the normal File Transfer Protocol (FTP) and Hypertext Transfer Protocol (HTTP) [9]. Furthermore, sequence data uploaded or downloaded by GBox can be readily processed using data processing tools implemented with a graphical user interface in a cloud computing service called Bio-Express for large-scale biological data analysis. This cloud computing service is equipped with data analysis utilities for various sequence data types, such as genomes, transcriptomes, epigenomes, metagenomes, and single-cell genomes [8,9]. This seamless coupling of KoNA, GBox, and Bio-Express, each having a user-friendly interface, facilitates the data experience, including submission, access, and reuse of raw nucleotide sequences. The current quota per user of Bio-Express is 100 central processing unit (CPU) cores, 400 GB of memory, and 1 TB of storage.

### Data statistics and contents

As of July 2022, 360 BioProjects, 653 BioSample Groups, 27,209 BioSamples, 666 Submissions, 27,634 Experiments, and 30,423 Runs have been deposited in the KRA. The 666 Submissions in the KRA database contained 477 TB of raw sequence data. Currently, KRA has a lower volume of deposited data than NCBI’s SRA (approximately 20 PB in September 2021) [1] or GSA and GSA-Human (approximately 9 PB in June 2021) [11]. However, the data volume in the KRA is expected to be comparable to that of other databases in the coming years, as the KRA collects a substantial portion of raw sequence data generated from Korean government-funded research projects.

**Figure 3:**
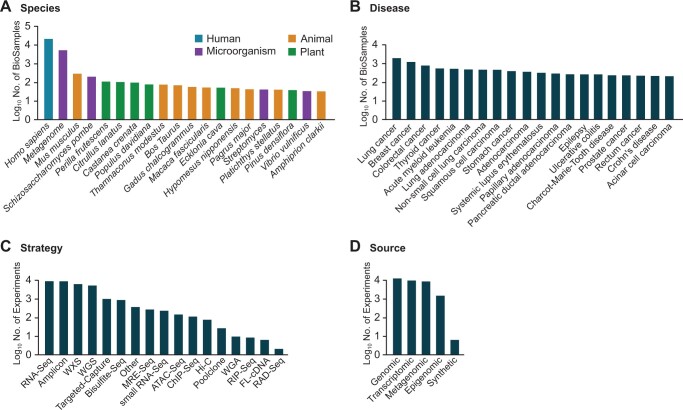
Summary of data statistics **A**. Top 20 species in KoNA with respect to the number of BioSamples. Each color denotes the sample type (human, animal, microorganism, and plant) that the species belongs to. **B**. The top 20 diseases in KoNA with respect to the number of human BioSamples. **C**. The number of strategies for generating raw NGS data. **D**. The number of sources used for sequencing experiments. RNA-Seq, RNA sequencing; WXS, whole-exome sequencing; WGS, whole-genome sequencing; Targeted-Capture, target capture sequencing; Bisulfite-Seq, bisulfite sequencing; MRE-Seq, methylation sensitive restriction enzyme sequencing; small RNA-Seq, small RNA sequencing; ATAC-Seq, assay of transposase accessible chromatin sequencing; ChIP-Seq, chromatin immunoprecipitation sequencing; Hi-C, high chromatin sequencing; Poolclone, pooled clone sequencing; WGA, whole-genome amplification; RIP-Seq, RNA immunoprecipitation sequencing; FL-cDNA, full-length cDNA sequencing; RAD-Seq, restriction site associated DNA sequencing.

Regarding the 360 BioProjects, more than 198 principal investigators participated in data deposition. Furthermore, the 27,209 BioSamples encompass 436 organisms, which correspond to 280 microbial, 62 animal, 93 plant species, and one human species (*Homo sapiens*). Notably, 26,127 of the 27,207 BioSamples (96.0%) belong to the top 20 species with the largest number of BioSamples; 19,788 (72.7%) and 4932 (18.1%) originate from humans and metagenomes, respectively (**Figure 3**A). In total, 19,788 human BioSamples are annotated with more than 87 diseases. Among the top 20 diseases with the largest number of human BioSamples in KoNA, 17 of 20 diseases are cancer diseases (Figure 3B). Such data, which are provided with rich metadata after the quality control process by KoNA curators, may reveal current research trends in Korean government-funded research projects. In addition, RNA sequencing (RNA-Seq) is the most common library strategy (Figure 3C), and genomic source is the most common source (Figure 3D).

## Discussion

In this study, we introduced KoNA as a nucleotide sequence repository for K-BDS. Following the announcement of an initiative called the “Strategy on Biological Research Resource Big Data” by the Korean government in 2020, K-BDS was constructed to collect all types of biological data generated from government-funded research projects. KoNA is a subdivision of K-BDS, which specializes in nucleotide sequences. Currently, KoNA accepts NGS data submissions with a plan to extend it to other types of nucleotide sequences, such as assembled sequences and primer sequences. We also plan to improve the KGA in the K-BDS, which currently has only a few functions for access requests and approval of human data deposited in the KRA. Because the data collection of KoNA is to be expanded to all primary nucleotide data, KGA is also updated to manage all sequence data and paired clinical data.

Although we have successfully collected and shared domestic NGS data in Korea through the KoNA over the years, we are also aware of the limitations of not yet being aligned with the INSDC, the principal authority for the international sharing of nucleic acid data. To overcome this limitation and progress to an internationally recognized data resource, we recently attempted to build a close collaboration with the INSDC by further strengthening our existing collaboration with the DDBJ. Specifically, arrangements and preparations are underway to confirm the agreement of data models between KoNA and INSDC and to share the data in KoNA with INSDC through DDBJ.

To ensure the quality of the deposited data, expert curators at KOBIC perform data curation. They categorize data and metadata based on the research context to increase data findability and accessibility. Related BioProjects, BioSample Groups, and Submissions are categorized into collections based on the research context. For example, whole-genome sequencing (WGS) data from patients with lung cancer are automatically collected in terms of “WGS”, “lung cancer”, and “*homo sapiens*”. The KoNA website is under development to display these collections with attribute information. Such datasets enable users to access the preferred sets deposited in the KoNA and run advanced queries on the database by selecting from the previously extracted attribute information of the collection. Then, to enhance the usability of the collection sets, we plan to construct secondary databases such as 3DIV [12], iCSDB [13], ChimerDB [14], and GEMiCCL [15], which will provide useful information using processed NGS and other nucleotide sequences. All these additional developments currently underway or planned will satisfy the unmet need for a national sequence repository in Korea, provide datasets to researchers globally, and contribute to advances in genomics.

## Materials and methods

### Establishment of the integrated service for genomic big data in KOBIC

**Figure 4:**
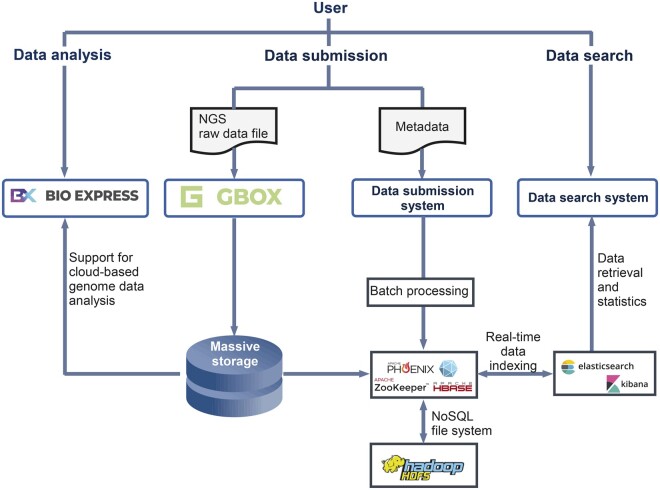
Schematic of the integrated service for genomic big data The interconnection between KoNA, GBox, and Bio-Express, supported by the HDFS, NoSQL-based database, and Elasticsearch, improves user convenience in data submission, transfer, retrieval, and reuse. HDFS, Hadoop Distributed File System.

To enhance data submission, transfer, retrieval, and reuse, an advanced integrated service for genomic big data was established by KOBIC by integrating Bio-Express, GBox, and NoSQL-based database architectures (**Figure 4**).

GBox, developed by KOBIC, provides fast data transfer and high-capacity cloud storage for integrated services. Two relay servers secure stability and enable about four times faster upload speed than Galaxy FTP (9.7 Mb/s and 1.8 Mb/s for GBox and Galaxy FTP, respectively). After transmission with GBox, the data are divided into blocks and stored in an Apache Hadoop file system [Hadoop Distributed File System (HDFS); version 2.7.1] to facilitate high-capacity cloud storage.

Data reuse is facilitated by supporting the analysis of genomic big data with Bio-Express, which is equipped with various bioinformatics analysis pipelines and uses Hadoop-based distributed and parallel processing for the fast analysis of big data. For example, for the variant calling process of 500 GB of WGS data with the Genome Analysis Toolkit (GATK) pipeline in the same hardware (Intel Xeon Gold 6132 CPU @ 2.60 GHz × 28 with 37 GB memory), the use of parallel computing in Bio-Express is approximately two times faster than in an ordinary setting.

As the backend database of the integrated service, the Apache HBase (version 2.0.5), a NoSQL database, with Apache ZooKeeper (version 3.6.0) and Phoenix was adopted on the HDFS. This NoSQL-based database enables effective storage and real-time retrieval of a large amount of data and metadata and supports flexible changes in data models by storing metadata in the JavaScript Object Notation (JSON) format, which allows a variable structure without a set schema. Moreover, the application of HDFS and Apache HBase in KoNA and Bio-Express facilitates the distributed and parallel processing of genomic big data in a high-performance computing cluster consisting of multiple computational nodes connected by the InfiniBand network. The KoNA database adopts Lucene-based Elasticsearch (version 6.3.1) [10] for data search and indexing and Kibana (version 6.3.1) for real-time statistical visualization of a large amount of genomic metadata.

### Implementation of the KoNA web service

The KoNA web service was implemented using Spring Boot, an application framework and inversion of the control container (http://www.springsource.org, version 2.2.4), and Thymeleaf, a server-side Java view template engine (version 3.0.12). All code was developed using Eclipse (http://www.eclipse.org), an integrated development environment for Java-based web applications. To provide stable web services, KoNA is hosted on a CentOS7 operating system with three servers: a Spring Boot-based web server, an Apache HBase server for database management, and a GBox server for fast file upload and download.

## Data Availability

KoNA is available at https://www.kobic.re.kr/kona/.

## CRediT author statement

**Gunhwan Ko:** Conceptualization, Software, Writing – original draft. **Jae Ho Lee:** Conceptualization, Investigation, Data curation, Writing – original draft, Visualization. **Young Mi Sim:** Investigation, Data curation. **Wangho Song:** Software. **Byung-Ha Yoon:** Software, Writing – original draft. **Iksu Byeon:** Resources. **Bang Hyuck Lee:** Resources. **Sang-Ok Kim:** Investigation. **Jinhyuk Choi:** Software, Investigation. **Insoo Jang:** Software. **Hyerin Kim:** Methodology. **Jin Ok Yang:** Methodology. **Kiwon Jang:** Methodology. **Sora Kim:** Methodology. **Jong-Hwan Kim:** Investigation. **Jongbum Jeon:** Investigation. **Jaeeun Jung:** Methodology. **Seungwoo Hwang:** Methodology, Writing – original draft, Writing – review & editing, Supervision. **Ji-Hwan Park:** Conceptualization, Writing – original draft, Writing – review & editing, Supervision. **Pan-Gyu Kim:** Conceptualization, Supervision. **Seon-Young Kim:** Conceptualization, Writing – original draft, Supervision, Funding acquisition. **Byungwook Lee:** Conceptualization, Writing – original draft, Writing – review & editing, Supervision, Project administration. All authors have read and approved the final manuscript.

## ORCID

0000-0002-3570-9074 (Gunhwan Ko)

0009-0003-1273-8256 (Jae Ho Lee)

0000-0003-2592-5408 (Young Mi Sim)

0000-0001-6992-9123 (Wangho Song)

0000-0002-5663-5000 (Byung-Ha Yoon)

0000-0002-7086-2111 (Iksu Byeon)

0009-0006-4927-0273 (Bang Hyuck Lee)

0000-0002-5526-798X (Sang-Ok Kim)

0000-0001-6434-6367 (Jinhyuk Choi)

0000-0003-0390-2715 (Insoo Jang)

0000-0003-4962-8272 (Hyerin Kim)

0000-0001-8388-6692 (Jin Ok Yang)

0000-0002-3789-5667 (Kiwon Jang)

0000-0003-4711-5246 (Sora Kim)

0000-0002-9520-0771 (Jong-Hwan Kim)

0000-0003-3533-1363 (Jongbum Jeon)

0000-0001-5179-4603 (Jaeeun Jung)

0000-0002-6415-9913 (Seungwoo Hwang)

0000-0002-6988-6239 (Ji-Hwan Park)

0000-0002-6069-1611 (Pan-Gyu Kim)

0000-0002-1030-7730 (Seon-Young Kim)

0000-0003-2083-8508 (Byungwook Lee)
